# Regime specific spillover across cryptocurrencies and the role of COVID-19

**DOI:** 10.1186/s40854-020-00210-4

**Published:** 2021-01-06

**Authors:** Syed Jawad Hussain Shahzad, Elie Bouri, Sang Hoon Kang, Tareq Saeed

**Affiliations:** 1 Montpellier Business School, Montpellier, France; 2 South Ural State University, Chelyabinsk, Russian Federation; 3 Adnan Kassar School of Business, Lebanese American University, Beirut, Lebanon; 4 Department of Business Administration, Pusan National University, Busan, South Korea; 5 Nonlinear Analysis and Applied Mathematics (NAAM)-Research Group, Department of Mathematics, Faculty of Science, King Abdulaziz University, Jeddah, Saudi Arabia

**Keywords:** Regime-switching, Volatility regimes, Spillovers, Connectedness, Cryptocurrencies, COVID-19

## Abstract

The aim of this study is to examine the daily return spillover among 18 cryptocurrencies under low and high volatility regimes, while considering three pricing factors and the effect of the COVID-19 outbreak. To do so, we apply a Markov regime-switching (MS) vector autoregressive with exogenous variables (VARX) model to a daily dataset from 25-July-2016 to 1-April-2020. The results indicate various patterns of spillover in high and low volatility regimes, especially during the COVID-19 outbreak. The total spillover index varies with time and abruptly intensifies following the outbreak of COVID-19, especially in the high volatility regime. Notably, the network analysis reveals further evidence of much higher spillovers in the high volatility regime during the COVID-19 outbreak, which is consistent with the notion of contagion during stress periods.

## Introduction

Following the appearance of Bitcoin in early 2009 and the ingenuity of its decentralized technology, called blockchain, several altcoins were released, making the cryptocurrency markets a new digital asset class worthy of consideration for investors, regulators, and academics. Earlier studies look at the technological and legal aspects of Bitcoin and other leading cryptocurrencies (Folkinshteyn and Lennon 2016), while later studies consider the economics and finance (e.g., Bouri et al. 2017; Ji et al. 2018; Shahzad et al. 2019; Kristjanpoller et al. 2020). They mainly focus on price formation by examining factors such as attractiveness (Kristoufek 2013), trading volume (Balcilar et al. 2017), and economic and financial variables.^1^ However, if cryptocurrencies represent a separate asset class as it is often argued, it is informative to study their inter-price dynamics for the sake of traders and portfolio managers who can exploit evidence concerning how the price of one cryptocurrency can affect the prices of other cryptocurrencies while making inferences about price predictability in the highly controversial cryptocurrency markets (Koutmos 2018; Corbet et al. 2018; Kumar and Ajaz 2019; Zięba et al. 2019; Ji et al. 2019).

In this regard, the existing literature covering the return connectedness among Bitcoin and other leading cryptocurrencies remains centred around the measurement of spillovers independent of any regime, or mostly based on one regime (Koutmos 2018; Ji et al. 2019; Zięba et al. 2019) and uses raw return data without accounting for the three factors of Shen et al. (2020a). However, it is often argued that return spillovers become stronger or more intense during unstable periods than calm periods, which necessitates the possibility of considering two volatility regimes—high and low. In fact, the dynamics of spillover depend on two distinct regimes, a high volatility regime during crisis periods and a low volatility regime during stable periods (BenSaïda et al. 2018; Reboredo and Ugolini 2020). While this has been applied to conventional assets and financial markets (BenSaïda et al. 2018; Reboredo and Ugolini 2020), it remains understudied in the cryptocurrency markets.

In this paper, we study the dynamics of return spillovers among cryptocurrencies with respect to global risk factors (e.g., COVID-19) under two volatility regimes (high and low) identified using a Markov regime-switching (MS) vector autoregressive (VAR) with exogenous variables model (i.e., MS-VARX). There is a high degree of flexibility in exploiting the power of Markov switching models for detecting abrupt regime shifts without specifying or fixing the shifts a priori. Our decision to use a VARX model instead of the standard MS-VAR model is motivated by the three factors (excess market returns, size, and reversal factor) identified by Shen et al. (2020a).

It is plausible that returnspillovers in cryptocurrency markets exhibit various patterns in response to the extreme random events manifest in various volatile regimes (Chaim and Laurini 2019).^2^ This is very relevant to the COVID-19 outbreak, which represents an extreme shock potentially shaping the dynamics of return spillovers among cryptocurrencies, and which represents an opportunity to study return spillovers among cryptocurrencies during a very stressful period. In the onset of the COVID-19 pandemic in early 2020, economic activities freezed, uncertainty spiked, and global financial markets tumbled. As a response, many central banks around the globe announced an unconventional monetary policy (i.e., quantitative easing) as an antidote to a deteriorating economic performance. Accordingly, there was a fear that fiat currencies will most likely lose value due to the quantitative easing, which makes cryptocurrencies under the spot again as a potential competitor. Given that and under the view that cryptocurrencies represent a new asset class, market participants would benefit from a refined understanding of the return spillovers among leading cryptocurrencies under the unprecedented turbulence of the COVID-19 outbreak.

Our analyses contribute to the existing literature on several fronts. Firstly, we use a sample spanning 25-July-2016 to 1-April-2020, covering the COVID-19 outbreak period during which financial markets crumbled (Gupta et al. 2021). This allows us to study return spillovers among cryptocurrencies during a very stressful time that represents the first global economic and financial catastrophe that occurred throughout the short existence of cryptocurrencies. Secondly, as the dynamics of return spillovers may depend on the regime, we incorporate MS within the VAR model of Diebold and Yilmaz (2012, 2014) and study return spillovers in the cryptocurrency markets under high and low volatility regimes. This represents an extension to the academic literature on regime-switching spillovers, which mainly concentrates on conventional assets such as equities (e.g., BenSaïda et al. 2018) but remains embryonic in the cryptocurrency markets. The few studies examining return spillovers in the cryptocurrency markets apply a single regime model (Koutmos 2018; Ji et al. 2019; Zięba et al. 2019). Thirdly, instead of incorporating only a MS-VAR model into the approach of Diebold and Yilmaz (2012), we also incorporate a MS-VARX model. In doing so, we account for the three-factor pricing model of Shen et al. (2020a) which is able to outperform the CAPM model in the cryptocurrency markets and thus extends the literature dealing with spillovers in the cryptocurrency markets based on raw return data (e.g., Koutmos 2018; Ji et al. 2019; Zięba et al. 2019). Overall, our paper is very pertinent to the academic debate about the cryptocurrency markets during the COVID-19 outbreak (Conlon and McGee 2020; Chen et al. 2020; Corbet et al. 2020; Conlon et al. 2020; Goodell and Goutte 2020; Dutta et al. 2020) and how the dynamics of return spillovers among cryptocurrencies change in regard to market downturns or crashes.

The empirical analyses show evidence of intensified return spillovers in the last 3 months of our sample period, belonging to the high volatility regime during the COVID-19 outbreak, revealing important aspects of the network of return spillovers across leading cryptocurrencies under various volatility states, and that the COVID-19 risk factors intensify that network. The overall results argue in favour of a fundamental breakdown in the return linkages.

This paper is structured as follows. “Literature reviews” section of this paper reviews the related literature; “Methodology” section provides the methods; “Empirical results” section presents the data and empirical results; and “Conclusion” section concludes.

## Literature reviews

The existing literature dealing with market linkages among cryptocurrencies is growing. It employs GARCH-based models, Granger causality tests, wavelets, and cointegration analyses Katsiampa et al. (2019b) use a bivariate BEKK-GARCH model on three pairs of cryptocurrencies (Bitcoin–Ether, Bitcoin–Litecoin, and Litecoin–Ether) and study the volatility dynamics’ conditional correlations. They show evidence of two-way volatility flows between for all three pairs and indicate that the three cryptocurrencies move in unison. They find evidence of two-way return flows between Bitcoin and both Ether and Litecoin, and a one-way flow from Ether to Litecoin. Using hourly data, Katsiampa et al. (2019a) apply symmetric and asymmetric multivariate GARCH models to examine the interactions of volatilities of eight cryptocurrencies. They show that Bitcoin is not the dominant cryptocurrency although its shocks on other cryptocurrencies are the longest lasting. Kumar and Ajaz (2019) apply wavelet-based methods and conclude that Bitcoin is the main driver of cryptocurrency prices. Using a Granger causality framework, Bouri et al. (2019a) study volatility linkages in the frequency domain and highlight the importance of large cryptocurrencies, other than Bitcoin. In another study, Bouri et al. (2019b) test for jumps in GARCH models and indicate that Bitcoin and 11 large and small altcoins exhibit jumps and co-jumps in their price process. They also show that Bitcoin and altcoins such as Ethereum and Ripple are important players in the cryptocurrency markets. Ciaian and Rajcaniova (2018) apply cointegration models and show significant evidence of interdependence between bitcoin and several altcoins that is mostly stronger in the short term.

Previous studies also apply the Diebold and Yilmaz (2012, 2014) models to static and dynamic connectedness in cryptocurrency markets. Koutmos (2018) focuses on Bitcoin and 17 large altcoins and finds that the connectedness measure varies with time and that Bitcoin and the altcoins examined are interconnected, with Bitcoin being a pivot in the network for return and volatility connectedness. Considering the cases of Bitcoin, Ripple, and Litecoin, Corbet et al. (2018) study return and volatility linkages and show that return shocks are mainly transmitted from Bitcoin to Ripple and Litecoin and that the return and volatility linkages between Ripple and Litecoin are strong. For the volatility linkages, they show that Litecoin and Ripple have a significant influence on Bitcoin, which is not in line with the findings in Koutmos (2018). Further results from Corbet et al. (2018) point to the isolation of the three cryptocurrencies under study from the global financial system, which suggests their ability to diversify the risk of conventional assets such as equities. Yi et al. (2018) examine the network of volatility among large cryptocurrencies and find that Bitcoin transmits its volatility to many cryptocurrencies, which makes it a dominant player in the network. Zięba et al. (2019) apply VAR models and a minimum spanning tree (MST) to the returns of Bitcoin and several other large altcoins, and show that Bitcoin is quite segmented from the other altcoins. Examining returns and volatility linkages, Ji et al. (2019) consider the case of Bitcoin and five other leading altcoins, highlighting the importance of Litecoin and Bitcoin to the network of return spillovers and the centrality of Bitcoin to the network of volatility spillovers. Later evidence contradicts Corbet et al. (2018) but confirms the earlier findings of Koutmos (2018). Ji et al. (2019) find that Dash is particularly segmented from Bitcoin and the rest of the altcoins under study, suggesting its potential as a diversifier. Notably, Ji et al. (2019) indicate that negative return connectedness is larger than positive return connectedness and that Ripple and Ethereum are the main receivers of negative-return shocks whereas Ethereum and Dash are marginal receivers of positive-return shocks. Qiao et al. (2020) employ wavelet coherence and correlation-based network to study the interdependence of the returns and volatility of cryptocurrencies. They find that Bitcoin and other cryptocurrencies are positively correlated at medium and high frequencies, whereas Bitcoin leads other cryptocurrencies at low frequencies. Furthermore, they indicate that the hedging effect of Bitcoin for other cryptocurrencies is time–frequency dependent. Qureshi et al. (2020) focus on the dynamic multiscale interdependencies among leading cryptocurrencies. They show high levels of dependency from 2016 to 2018 at daily frequency scales and that Ripple and Ethereum are trivial origins of market contagion. Further results indicate that the coherence fluctuates at higher frequencies, but it is significantly stable at lower frequencies. Antonakakis et al. (2019) examine connectedness measures among leading cryptocurrencies using a time-varying parameter factor augmented VAR (TVP-FAVAR) model. They indicate a time-variation in the connectedness measures and show that Bitcoin is the most important transmitter of shocks in the cryptocurrency markets, followed by Ethereum.

To address the impact of COVID-19 on thecryptocurrency market, empirical studies have investigated the contagion between the pandemic and the financial markets (Akhtaruzzaman et al. 2020; Azimli 2020; Baker et al. 2020; Bouri et al. 2020b; Goodell and Goutte 2020; Topcu and Gulal 2020), opportunities for portfolio diversification (Akhtaruzzaman et al. 2020; Corbet et al. 2020; Conlon and McGee 2020; Yoshino et al. 2020), and commovment between cryptocurrencies (Yarovaya et al. 2020). Due to the outbreak of the COVID-19, portfolio investors continually seek an alternative asset to protect the extreme downside risk of financial assets.^3^ Cryptocurrencies have attracted the attention of many investors, as they offer the benefit of a diversifier, a hedge asset or a safe haven asset against the downside risk of traditional investments (Bouri et al. 2017).

The above-mentioned literature neglects the possibility of having various volatility states—high and low—in the network of connectedness among cryptocurrency returns. As argued by BenSaïda et al. (2018), there is economic merit to incorporating Markov switching within the generalized vector autoregressive (VAR) model of Diebold and Yilmaz (2012, 2014), given that return spillovers among markets are stronger and more intense during unstable periods than calm periods. Therefore, the dynamics of return spillovers are regime dependent and any analysis of connectedness that neglects the shift in the volatility regimes can lead to spurious findings. In this paper, we study return spillovers among leading cryptocurrencies while capturing shifts in regimes due to the COVID-19 outbreak, sudden events, or changes in market conditions. Our current paper is related to a newly rising strand of literature that deals with the role of Bitcoin and other cryptocurrencies against conventional assets during the COVID-19 outbreak (Conlon and McGee 2020; Chen et al. 2020; Corbet et al. 2020; Conlon et al. 2020; Goodell and Goutte 2020; Dutta et al. 2020). However, it differs in several aspects. Firstly, it focuses on the return spillovers among cryptocurrencies while considering two volatility regimes, high and low, which is an unexplored research subject in the controversial cryptocurrency markets. Secondly, it accounts for the three factors (excess market returns, size, and reversal factor) identified by Shen et al. (2020a), which nicely extends previous studies that use raw return data (Koutmos 2018; Ji et al. 2019; Zięba et al. 2019).

## Methodology

We capture the changes in return spillovers with respect to global risk factors (e.g., COVID-19) under two volatility regimes (high and low) identified using a Markov regime-switching (MS) vector autoregressive with exogenous variables (VARX) model. The VARX model accounts for the three factors identified by Shen et al. (2020a), which are excess market returns, size, and reversal factors.

### The MS-VARX model

Our MS-VARX model is specified as:

1 $$y_{t} {\mid }s_{t} = \nu_{k} + \mathop \sum \limits_{i = 1}^{p} \Phi_{k,i} y_{t - i} + \Psi_{k} X_{t} + u_{{s_{t} ,t}}$$

where $y_{t} = \left( {y_{1,t} , \ldots ,y_{n,t} } \right)^{^{\prime}}$ for t = 1,…, T; $\nu_{k}$ is a (n × 1) regime-dependent vector of intercepts; $\left\{ {{\Phi }_{k,i} } \right\}_{i = 1}^{p}$ are (n × n) state-dependent matrices, where Φ_k_,_p_ ≠ 0 and 0 represents the n × n null matrix; $X_{t}$ is the vector of three-factors (excess market returns, size, and reversal), which are inspired from the cryptocurrency model of Shen et al. (2020a); and $u_{{s_{t} ,t}}$ is a vector of errors.

To consider the fact that each regimes might have different variances we set $u_{{s_{t} ,t}} = \Sigma_{k}\upvarepsilon _{t}$, where $\upvarepsilon _{t} \mathop \sim \limits^{i.i.d.} {\mathcal{N}}\left( {0_{n} ,{\text{I}}_{n} } \right)$, with ${\text{I}}_{n}$ denoting the identity matrix, and $0_{n}$ representing a $\left( {{\text{n}} \times 1} \right)$ vector of 0. Σ_k_, symbolizes a lower triangular $\left( {{\text{n}} \times {\text{n}}} \right)$ regime-dependent Cholesky factorization of the symmetric variance–covariance matrix denoted by Ω_k_. Therefore, we can write:

2 $${\varvec{y}}_{t} {\mid }s_{t} \sim {\mathcal{N}}\left( {{\varvec{\nu}}_{k} ,{\varvec{\varOmega}}_{k} } \right)$$

where each regime k = {1,…, K} is described by its own ν_k_, $\left\{ {{{\varvec{\Phi}}}_{k,i} } \right\}_{i = 1}^{p}$, and Ω_k_. The model considers the possibility of varying intercepts following the state of the market, dictated by the state variable {s_t_}. The autoregressive matrices $\left\{ {{{\varvec{\Phi}}}_{k,i} } \right\}_{i = 1}^{p}$ govern the intensity of spillovers across cryptocurrency variables, according to the regime. The Markov switching variance–covariance matrix Ω_k_ allows us to identify structural shocks in the residuals.

The state variable {s_t_} progresses in line with a discrete, homogeneous, and finite state irreducible first-order Markov chain with a transition probability matrix P. Each element of P represents the conditional probability of transitioning from regime *i* to regime *j*. Accordingly, we can write:

3 $$\begin{aligned} & {\varvec{P}} = \left( {\begin{array}{*{20}c} {p_{1,1} } & \cdots & {p_{1,K} } \\ \vdots & \ddots & \vdots \\ {p_{K,1} } & \cdots & {p_{K,K} } \\ \end{array} } \right) \\ & p_{i,j} = Pr\left( {s_{t} = j{\mid }s_{t - 1} = i} \right) \\ \end{aligned}$$

where the sum of each column of P is equal to 1. If we have two regimes, then the transition matrix is given by:

4 $${\varvec{P}} = \left( {\begin{array}{*{20}c} p & {1 - q} \\ {1 - p} & q \\ \end{array} } \right)$$

The unconditional probability π is the eigenvector of P, satisfying P π = π, and $1_{K}^{^{\prime}}$ π = 1, where 1_K_ is a (K × 1) column vector of 1. Accordingly:

5 $${\varvec{\pi}} = \left( {{\mathbf{A^{\prime}A}}} \right)^{ - 1} {\mathbf{A^{\prime}}}\left[ {\begin{array}{*{20}c} {0_{K} } \\ 1 \\ \end{array} } \right]$$

where 0_K_ is a (K × 1) column vector of 0, and:

6 $$\mathop {\mathbf{A}}\limits_{{\left( {K + 1} \right) \times K}} = \left[ {\begin{array}{*{20}c} {{\mathbf{I}}_{K} - P} \\ {1_{K}^{^{\prime}} } \\ \end{array} } \right]$$

In the presence of two regimes, we present the unconditional probabilities as:

7 $$\left\{ {\begin{array}{*{20}l} {\pi _{1} = \frac{{1 - q}}{{2 - p - q}}} \hfill \\ {\pi _{2} = \frac{{1 - p}}{{2 - p - q}}} \hfill \\ \end{array} } \right.$$

For the estimation method, the reader can refer to BenSaïda et al. (2018).^4^

### The concept of spillovers

The spillover measure of Diebold and Yilmaz (2014) is based on the forecast error variance decomposition from the VAR model. Notably, a generalized impulse response function is used, which does not require orthogonalization by Cholesky decomposition. Accordingly, the spillover index is invariant to ordering. Given the covariance-stationary model in Eq. (1) we present its vector moving average representation (see BenSaïda et al. 2018) as follows:

8 $${\varvec{y}}_{t} {\mid }s_{t} = {\varvec{\omega}}_{k} + \mathop \sum \limits_{j = 0}^{\infty } {\mathbf{A}}_{k,j} {\varvec{u}}_{{s_{t} ,t - j}}$$

where $${\mathbf{A}}_{k,j} = \sum\nolimits_{i = 1}^{p} {{{\varvec{\Phi}}}_{k,i} {\mathbf{A}}_{k,j - i} }$$, and $${\varvec{\omega}}_{k} = \left( {{\mathbf{I}}_{n} - \sum\nolimits_{i = 1}^{p} {{{\varvec{\Phi}}}_{k,i} } } \right)^{ - 1} {\varvec{\nu}}_{k}$$.

We define the generalized *H*-step ahead forecast error variance decomposition shares in each regime *k* as:

9 $$\theta_{k,ij}^{g} \left( h \right) = \frac{{\sigma_{k,jj}^{ - 1} \mathop \sum \nolimits_{l = 0}^{h - 1} \left( {{\varvec{e}}_{i}^{^{\prime}} {\mathbf{A}}_{k,l} {{\varvec{\Sigma}}}_{k} {\varvec{e}}_{j} } \right)^{2} }}{{\mathop \sum \nolimits_{l = 0}^{h - 1} \left( {{\varvec{e}}_{i}^{^{\prime}} {\mathbf{A}}_{k,l} {{\varvec{\Sigma}}}_{k} {\mathbf{A}}_{k,l}^{^{\prime}} {\varvec{e}}_{i} } \right)}}$$

where ${\upsigma }_{{k, jj{ }}}$ is the standard deviation of the error term of the *j*th equation, and $e_{i}$ is a vector with 1 on the *i*th element and 0 otherwise. Given the use of the generalized impulse response functions, it is important to normalize each entry of the variance decomposition matrix to ensure that each row sums up to 1 as:

10 $$\tilde{\theta }_{k,ij}^{g} \left( h \right) = \frac{{\theta_{k,ij}^{g} \left( h \right)}}{{\mathop \sum \nolimits_{j = 1}^{n} \theta_{k,ij}^{g} \left( h \right)}}$$

The total spillover index in regime k is given in Eq. (11). This measures the contribution of spillovers from volatility shocks among cryptocurrencies in the system to the total forecast error variance:

11 $$S_{k}^{g} \left( h \right) = \frac{1}{n}\mathop \sum \limits_{{\begin{array}{*{20}c} {i,j = 1} \\ {i \ne j} \\ \end{array} }}^{n} \tilde{\theta }_{k,ij}^{g} \left( h \right)$$

The directional spillovers received by cryptocurrency *i* from all other cryptocurrencies* j* is:

12 $$\mathop {S_{k}^{g} }\limits_{all \to i} \left( h \right) = \frac{1}{n}\mathop \sum \limits_{{\begin{array}{*{20}c} {j = 1} \\ {i \ne j} \\ \end{array} }}^{n} \tilde{\theta }_{k,ij}^{g} \left( h \right).$$

The directional spillovers transmitted by cryptocurrency *i* to all other cryptocurrencies *j* is:

13 $$\mathop {S_{k}^{g} }\limits_{i \to all} \left( h \right) = \frac{1}{n}\mathop \sum \limits_{{\begin{array}{*{20}c} {i = 1} \\ {i \ne j} \\ \end{array} }}^{n} \tilde{\theta }_{k,ij}^{g} \left( h \right).$$

The net spillover from cryptocurrency *i* to all other cryptocurrencies is:

14 $$S_{k,i}^{g} \left( h \right) = \mathop {S_{k}^{g} }\limits_{i \to all} \left( h \right) - \mathop {S_{k}^{g} }\limits_{all \to i} \left( h \right)$$

## Empirical results

### The dataset

This paper uses daily price data of 18 cryptocurrencies, Bitcoin (BTC), Etreum (ETH), Ripple (XRP), Litecoin (LTC), Monero (XMR), Stellar (XLM), Dash (DASH), Ethereum Classic (ETC), NEM (XEM), Dogecoin (DOGE), Decred (DCR), Lisk (LSK), Was (WAVES), MonaCoin (MONA), DigiByte (DGB), Steem (STEEM), Siacoin (SC), and DigixDAO (DGD). The data sample spans July 25th, 2016 to April 1st, 2020 which covers various financial, economic, and pandemic events. Our sample period covers various events that can shape investors and markets of cryptocurrencies. These include four categories, split, regulation, exchange, and hacking. Table 1 summarizes those events, their dates, and the sign (positive/negative) of their impacts. Regarding the data, they are extracted from https://coinmarketcap.com.^5^ We consider the three-factor pricing model proposed by Shen et al. (2020a), SMB representing the return spread of small minus large stocks, WML representing the equal-weight average of the returns for the two winner portfolios for a region minus the average of the returns for the two loser portfolios, and MKT representing the return spread between the capitalization weighted stock market. These daily factors are calculated using trading data of 1967 cryptocurrencies for the sampled period. Cryptocurrency price data are collected from https://coinmarketcap.com/. The T-Bill rate is used as a proxy for the risk-free asset and obtained through the US Department of the Treasury. The detailed procedure to compute these factors is given by Shen et al. (2020a).

**Table 1:** Selected events related to the cryptocurrency markets

Event description	Date	Category	Sign of the impact
Bitcoin splits into Bitcoin and Bitcoin Cash	August 1, 2017	Split	+
China shuts down all cryptocurrency exchanges	September 15, 2017	Regulation	−
CME, CBOE to Begin Bitcoin Futures Trading	December 1, 2017	Exchange	+
CBOE Bitcoin futures are launched	December 11, 2017	Exchange	+
Japanese cryptocurrency exchange loses more than $500 million to hackers	January 26, 2018	Hacking	−

We calculate the continuously compounded daily returns by taking the difference in the log values of two consecutive prices. Table 2 shows the summary statistics of the returns series for the 18 cryptocurrencies and three factors. In Panel A, the mean returns of all cryptocurrencies are positive except for STEEM. Importantly, the XRP returns exhibit the highest mean return among the cryptocurrencies. Looking at the standard deviation, DGB is the most volatile cryptocurrency, followed by STEEM, while BTC is the least volatile. The skewness coefficient values are positive for all series except BTC and ETH returns. The kurtosis coefficient value is largely above 3, the value of normal distributions, indicating leptokurtic behaviour. The Jarque–Bera test strongly rejects the normal distribution of returns. In Panel B, all factor returns show negative values with a low value of standard deviation. All factors show the non-normality of return series due to the values of skewness, kurtosis and J–B statistics. It is worth noting that all return series are stationary according to the unit root (ADF) and stationary (KPSS) test estimations.

**Table 2:** Descriptive statistics

	Symbol	Mean	Std. Dev	Skewness	Kurtosis	J–B	ADF	KPSS
*Cryptocurrencies*								
Bitcoin	BTC	0.171	4.256	− 0.961	16.578	10,554.4***	− 37.57***	0.345
Ethereum	ETH	0.176	5.773	− 0.418	12.639	5254.1***	− 37.44***	0.478
Ripple	XRP	0.247	7.430	2.791	42.046	87,315.3***	− 23.09***	0.348
Litecoin	LTC	0.168	6.059	0.818	14.595	7695.4_***_	− 36.94***	0.391
Monero	XMR	0.241	6.713	0.763	13.263	6042.6***	− 38.23***	0.563
Stellar	XLM	0.215	8.084	1.990	19.934	16,984.0***	− 33.47***	0.374
Dash	DASH	0.146	6.198	0.632	11.486	4131.2***	− 36.61***	0.599
Ethereum Classic	ETC	0.125	8.001	4.019	85.511	385,728.***	− 45.20***	0.313
NEM	XEM	0.125	7.398	2.404	32.522	50,212.8***	− 40.90***	0.515
Dogecoin	DOGE	0.150	6.425	0.654	14.679	7751.2***	− 34.89***	0.200
Decred	DCR	0.142	7.426	0.701	9.156	2237.3***	− 40.72***	0.501
Lisk	LSK	0.090	7.225	0.144	9.529	2397.3***	− 36.46***	0.349
Waves	WAVES	0.116	6.875	0.114	7.902	1351.4***	− 36.67***	0.392
MonaCoin	MONA	0.238	8.552	2.786	24.701	28,173.5***	− 34.59***	0.241
DigiByte	DGB	0.186	8.918	2.207	29.752	41,261.4***	− 37.19***	0.350
Steem	STEEM	− 0.227	8.723	0.869	12.367	5094.3***	− 35.86***	0.161
Siacoin	SC	0.049	8.196	0.633	12.257	4899.8***	− 36.93***	0.288
DigixDAO	DGD	0.075	7.429	0.142	10.750	3375.9***	− 39.91***	0.297
*Factors*								
Size factor	SMB	− 0.004	0.038	0.475	6.833	875.4***	− 41.13***	0.277
Momentum	WML	− 0.100	0.042	− 0.653	5.905	569.6***	− 9.82***	0.958
*Market*								
(equally weighted)	MKT	− 0.005	0.045	− 1.128	11.180	4041.3***	− 11.42***	1.250

Descriptive statistics of price returns are reported over a period of 1347 trading days from 25-July-2016 to 1-April-2020. Descriptive statistics for the factors are reported in basis points for the levels of the series

***indicates significance at 1% level

**Fig. 1:**
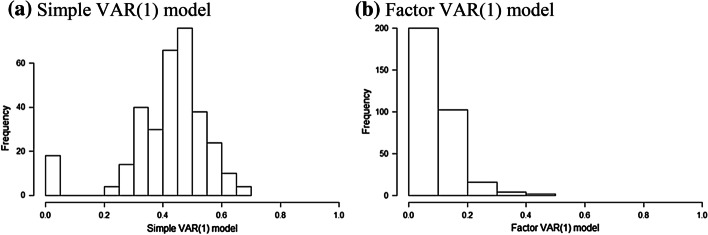
Comparison of absolute residual correlations, with and without factors. *Notes* The histograms show the distribution of the absolute pairwise correlations between the residuals of the simple VAR(1) model and our factor VAR(1) model evaluated by OLS

Before we present the main results of return spillovers, we evaluate the adequacy of our three-factors. To this end, we follow Ando et al. (2018) and compare the residual cross-correlations arising from the simple VAR(1) model to those arising from our three-factor VAR(1) model. Based on the assumption that the cross-sectional correlation of the VAR residuals is driven by a finite number of common factors, we purge the common component from the VAR residuals. This allows us to isolate the idiosyncratic shock to each cryptocurrency and reduces the likelihood of failure to account for sources of common variation which may generate substantial biases. Specifically, an omitted common factor upwardly biases the estimated spillover if the proportion of the forecast error variance is attributed to one or more of the endogenous variables instead of that common factor. Comparing Fig. 1b and a, it is clear that a substantial amount of the correlation among the residuals is removed when the factors are considered. Specifically, the factor VAR(1) model indicates that almost 95% of the pairwise correlations are weaker than 0.2 in absolute value, which points to the adequacy of our factors. This finding indicates that the residuals of the factor VAR(1) model are cross-sectionally uncorrelated.

### Single regime spillover

Table 3 provides a matrix of the directional spillovers across the 18 cryptocurrency markets. This table shows the directional spillover of a market to other markets (row titled “To”) and from other markets to that market (column titled “From”). The bottom row of Table 3 shows whether each market is a net receiver or net contributor to other markets.

**Table 3:** Directional spillovers under single normal regime—full sample

	BTC	ETH	XRP	LTC	XMR	XLM	DASH	ETC	XEM	DOGE	DCR	LSK	WAVES	MONA	DGB	STEEM	SC	DGD	From
BTC	82.23	1.32	0.49	2.68	1.14	0.77	1.01	0.39	1.39	0.17	0.56	0.84	0.10	1.72	1.51	0.42	2.72	0.55	17.77
ETH	0.56	69.40	0.68	3.30	3.84	1.09	5.16	3.59	2.16	0.24	0.68	1.68	0.88	0.34	0.42	0.11	1.30	4.57	30.60
XRP	0.48	0.69	74.50	2.10	0.67	15.03	0.25	0.15	1.28	2.89	0.05	0.07	0.24	0.12	0.69	0.38	0.25	0.17	25.50
LTC	2.55	3.62	1.59	72.22	2.14	1.44	2.51	3.82	1.65	2.55	0.71	0.83	0.34	0.60	0.48	0.15	1.11	1.70	27.78
XMR	1.57	3.99	1.05	2.14	72.24	3.15	7.24	0.75	1.13	0.20	0.62	0.90	1.22	0.52	0.78	0.54	0.80	1.15	27.77
XLM	0.66	0.76	13.56	1.10	2.24	68.95	0.10	0.35	2.21	3.43	0.16	0.40	0.33	0.07	2.35	1.41	1.62	0.27	31.05
DASH	0.75	4.95	0.22	2.56	7.05	0.13	70.45	3.06	1.55	0.12	2.38	2.59	0.50	0.72	0.32	0.67	1.25	0.74	29.55
ETC	0.34	3.62	0.22	4.07	0.68	0.70	2.65	80.37	0.84	0.80	0.28	2.05	0.03	0.31	0.12	0.14	1.45	1.32	19.64
XEM	0.96	2.45	1.47	1.61	1.38	2.52	1.62	1.08	76.30	0.78	0.92	3.31	0.37	0.91	0.41	1.67	1.26	0.96	23.70
DOGE	0.02	0.30	2.93	2.74	0.22	4.13	0.33	0.73	0.77	77.20	0.12	0.43	0.03	0.01	3.88	0.67	5.30	0.19	22.80
DCR	0.05	0.57	0.06	0.87	0.61	0.18	3.28	0.04	1.15	0.11	85.06	1.05	0.64	0.53	1.25	1.86	2.30	0.40	14.94
LSK	0.38	1.86	0.25	0.79	1.30	0.64	2.93	2.06	2.92	0.29	0.94	76.65	2.17	1.00	0.43	1.18	2.45	1.75	23.35
WAVES	0.10	1.02	0.07	0.60	1.16	0.51	0.44	0.07	0.42	0.07	0.70	2.48	89.02	0.01	0.82	0.76	1.31	0.44	10.98
MONA	0.34	0.51	0.13	0.76	0.59	0.10	0.96	0.42	0.98	0.05	0.46	0.94	0.02	92.56	0.09	0.32	0.36	0.41	7.44
DGB	0.70	0.11	1.78	0.41	0.22	3.13	0.10	0.09	0.32	3.23	1.61	0.49	0.67	0.16	74.30	0.71	11.72	0.25	25.70
STEEM	0.09	0.17	0.46	0.14	0.70	3.14	0.94	0.28	1.02	0.40	1.90	1.23	0.76	0.31	1.04	86.27	0.97	0.17	13.73
SC	0.59	1.26	0.13	0.56	0.59	1.72	1.15	1.04	1.16	4.71	1.47	2.20	0.72	0.59	11.36	1.02	69.13	0.59	30.87
DGD	0.50	5.42	0.22	2.10	1.30	0.31	1.03	1.39	0.83	0.01	0.43	1.83	0.27	0.49	0.12	0.12	0.57	83.07	16.93
To	10.65	32.64	25.32	28.53	25.82	38.69	31.73	19.32	21.79	20.05	13.99	23.32	9.29	8.40	26.05	12.14	36.73	15.63	Total
Net	− 7.12	2.04	− 0.18	0.75	− 1.94	7.64	2.19	− 0.31	− 1.92	− 2.75	− 0.96	− 0.03	− 1.69	0.97	0.34	− 1.59	5.86	− 1.30	22.23

This table shows connectedness across the cryptocurrencies computed using the approach of Diebold and Yilmaz (2012). “To” and “From” mean direction spillover to and from other markets to a specific currency. “Net” is the difference between “To” and “From”. A currency is a receiver if “Net” is negative and a transmitter of spillover if “Net” is positive

In the “To” row in Table 3, XLM is the largest contributor of shocks to the other markets with a contribution of 38.69%, followed by SC (36.73%), ETH (32.64%), and DASH (31.73%). In the “From” column, XLM is also the largest recipient of spillovers, with a contribution of 31.05%, followed by SC (30.87%), ETH (30.60%), LTC (27.78%), and XMR (27.77%). In terms of net spillovers (“To”–“From”), XLM is the largest net transmitter of spillovers, with a net value of 7.64%, followed by SC (5.86%), DASH (2.19%), and ETH (2.04%). Conversely, the largest net recipient of spillovers is BTC (− 7.12%) followed by DOGE (− 2.75%), XMR (− 1.94%), XEM (− 1.92%), WAVES (− 1.69%), and STEEM (− 1.59%). Therefore, XLM seems to play a dominant role in the spillover connectedness across cryptocurrency markets. As for the total spillover index, it is only 22.23%.

**Fig. 2:**
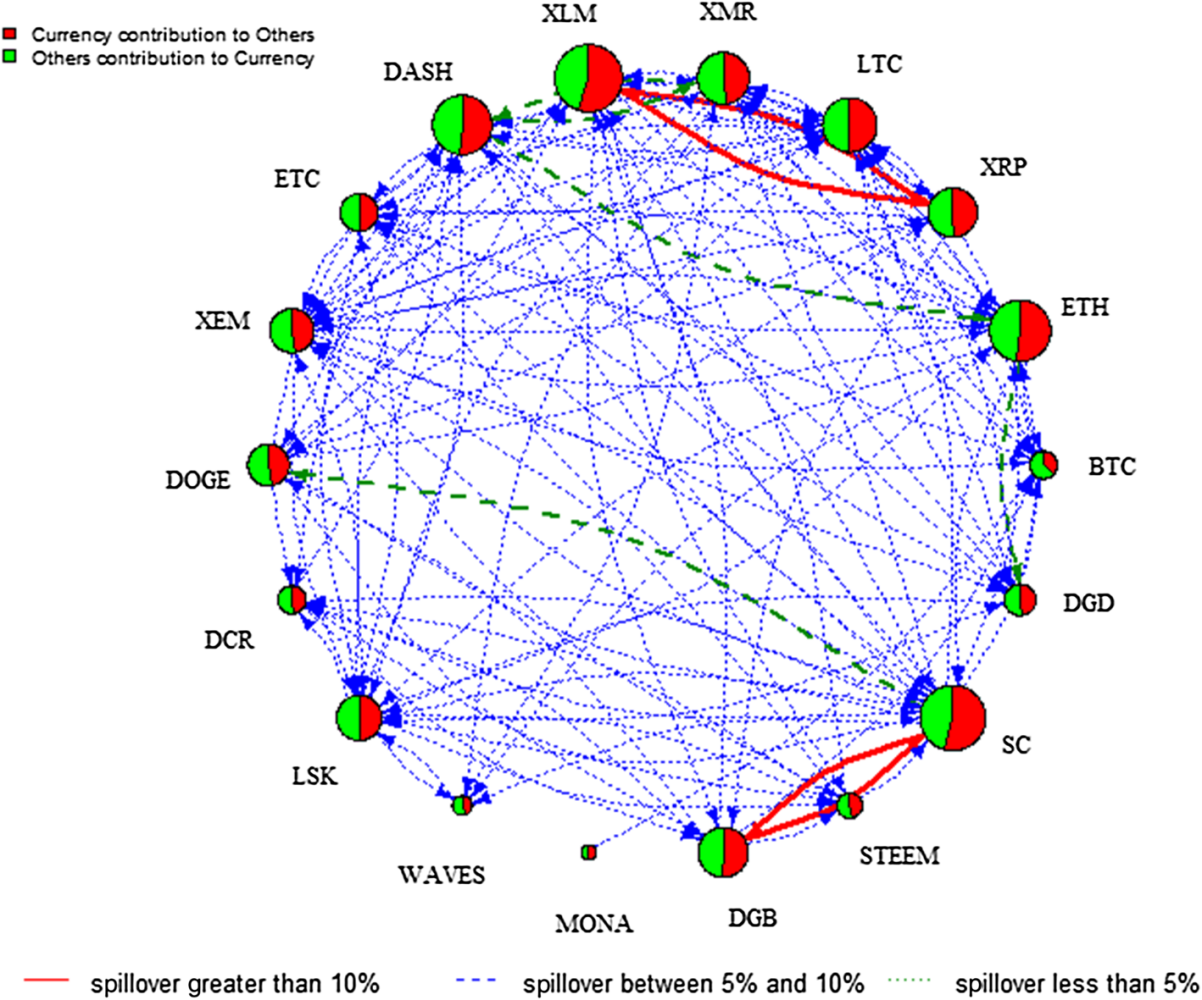
DY Spillover—without Regime Switching. *Notes*: This network graph illustrates the degree of total connectedness in a system that consists of the 18 cryptocurrencies over the full sample period. Total connectedness is measured using the Diebold–Yilmaz framework. The size of the node shows the magnitude of the contribution of each variable to system connectedness, while the colour indicates the origin of connectedness. In particular, red implies a contribution from the variable under consideration to the other variables of the system and green means a contribution from the other variables to the variable under analysis. The colour and thickness of edges refers to the strength of the connectedness. Specifically, arrows in red full lines indicate that the magnitude of the connectedness is greater than 10%, arrows in green dashed lines imply that the strength of the connectedness is between 5 and 10%, blue dotted lines are associated with connectedness between 1 and 5%. Finally, connectedness lower than the 1% is not reported to preserve clarity in the figure

Figure 2 visualizes the network of the pairwise directional connectedness across the 18 cryptocurrency markets over the full sample period, reported in Table 3. Note that the size of each node in the network relating the set of markets is determined by both the contribution in terms of the effect of each market on other markets (the sum of the coefficients in each column excluding own-market effects) and of other markets on any particular market (the sum of the coefficients in each row excluding own-market effects). The red colouring implies the contribution from the variable under consideration to the other cryptocurrencies of the system, whereas green indicates the contribution from the other cryptocurrencies to the cryptocurrency under analysis. The thickness of the edges refers to the strength of the connectedness. In Fig. 2 we can observe a bi-directional pairwise connectedness across most of the 18 cryptocurrency markets. In particular, XLM has strong connectedness (red edge) with XRP and other cryptocurrencies. Similarly, SC has a strong connectedness with DGB and other cryptocurrencies. A bi-directional spillover exists between DASH and XMR, and a significant spillover is shown from ETH to both DASH and DGD, and from SC to DOGE.

### Switching regime spillover

**Fig. 3:**
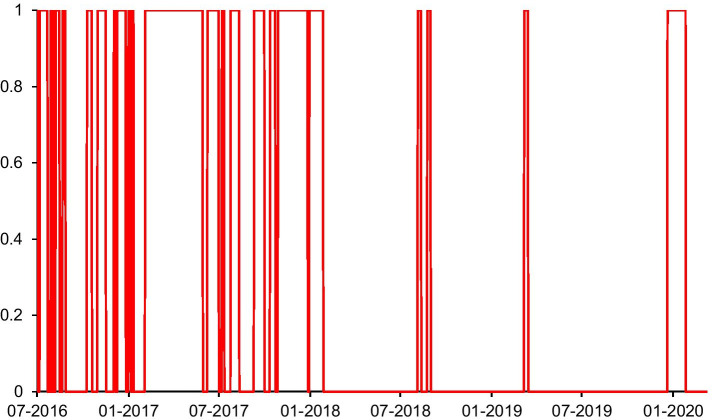
Smoothed probabilities of intense spillover (Regime 2)

Recent studies argue that the dynamics of spillover depend on two distinct regimes, a high volatility regime during crisis periods, and a low volatility regime during stable periods (BenSaïda et al. 2018; Reboredo and Ugolini 2020). In order to identify the two distinct regimes, we estimate a MS-VAR(1) model and differentiate between regime 1 and regime 2. Figure 3 shows several high volatility regimes with periods of intense spillover, during which the probabilities of being in regime 2 are nearly 1. We see high volatility regimes based on external shocks such as the rapid growth of cryptocurrency markets during 2016–2018 and the COVID-19 period during early 2020.

**Fig. 4:**
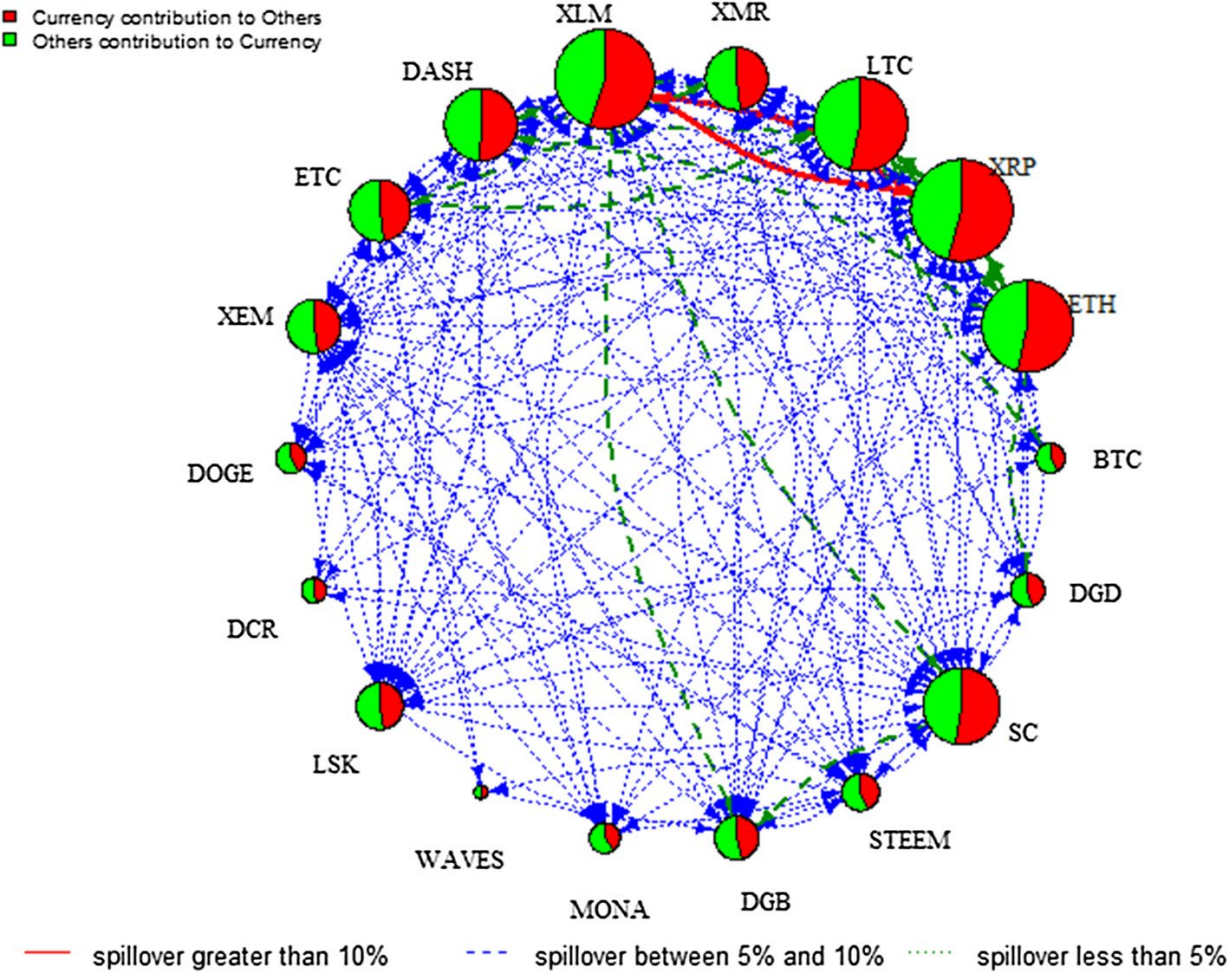
DY spillover network—low volatility regime. *Note*: See notes to Fig. 2

**Fig. 5:**
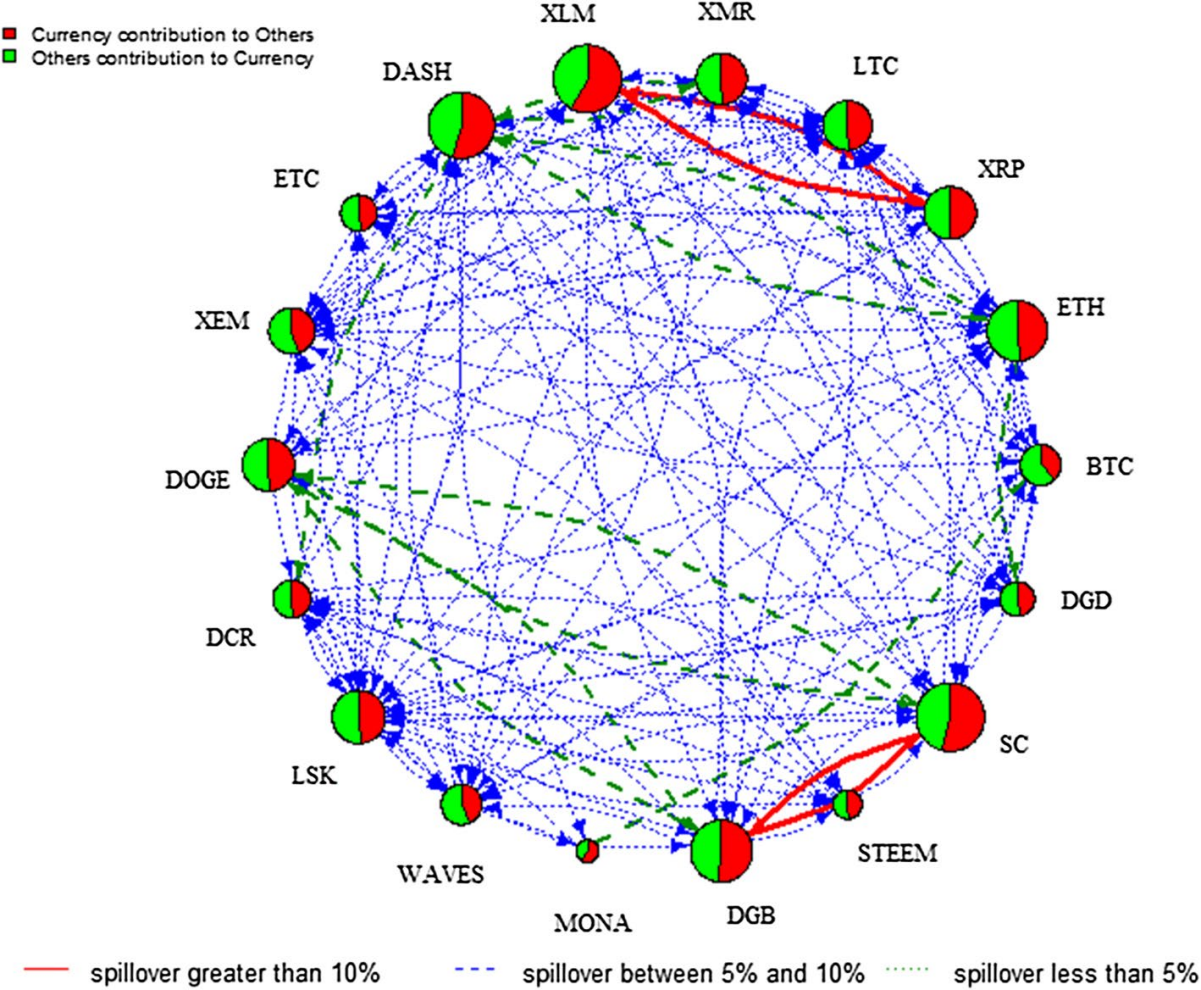
DY Spillover network—high volatility regime. *Note*: See notes to Fig. 2

Analysing these regimes provides in-depth knowledge of the change of the direction of spillover between the two regimes. Figures 4 and 5 plot the spillover network across the 18 cryptocurrency markets in low and high volatility regimes, respectively.^6^ Comparing the two regimes, the total spillover index is 25.70% in the low volatility regime compared to 29.43% in the high volatility regime. The node size of the spillover network in the low volatility regime is relatively larger than that in the high volatility regime. Specifically, it is evident that XRP, XLM, LTC, and ETH are more strongly connected to others in the low volatility regime, implying that these cryptocurrencies dominate the spillover in stable periods. Notably, the role of BTC is less important, which contradicts with Kumar and Ajaz (2019). However, the result is generally in line with Zięba et al. (2019) who highlight the importance of smaller cryptocurrencies to the network of return shocks due to the specificity of the supply mechanism of those cryptocurrencies. In addition, XLM is the largest contributor to others in both regimes, indicating that XLM is a hub market of the information spillover network. Compared to the network in Fig. 2, we can see pairwise spillover between XLM and XRP in both regimes and between SC and DGB in the high regime only. These figures show that the directional spillover effect is sensitive to the state of the volatility regime given that the spillover effect is more pronounced and concentrated among fewer cryptocurrencies in the low volatility regime.

**Fig. 6:**
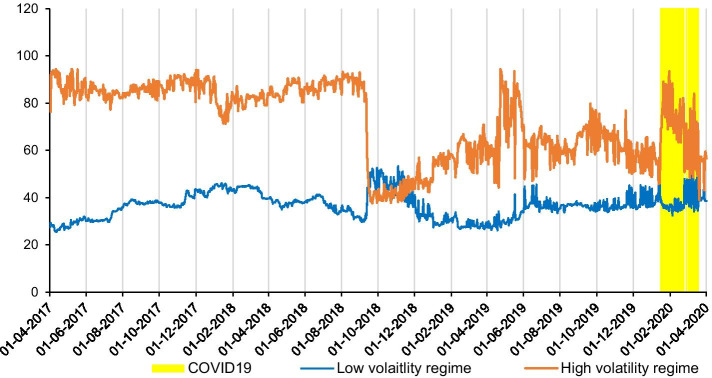
Total return spillovers. *Note*: The rolling window total spillover indices are based on 250 days rolling window, lag = 1 (based on SIC) and a forecast horizon of 12 days

Given the time-varying total spillovers among the 18 cryptocurrencies in the low and high volatility regimes, Fig. 6 shows a significant structural change of spillover in late 2018 when the market capitalization of Bitcoin fell below $100 billion and the price of one Bitcoin plunged below $4000 after losing almost one-third of its value in a few days. Before that period, the dynamics of total spillover are relatively smooth and consistent between the high and low volatility regimes. However, we see a sharp drop of total spillover in the high volatility regime and the convergence of both total spillovers during that period. Subsequently, the total spillover in the high volatility regime restarts upward, and, more interestingly, in the high volatility regime, a sudden increase in total spillover is observed in the COVID-19 outbreak period^7^ which intensifies the magnitude of spillover in the high volatility regime. This finding indicates that the dynamics of the total spillover index rapidly react to external shocks, which concords with previous findings (e.g., Antonakakis et al. 2019; Ji et al. 2019). Compared to the literature on contagion which highlights strong and abrupt changes in market linkages (Baele and Inghelbrecht 2010), our findings indicate evidence of contagion in response to external shocks such as the COVID-19 outbreak. This finding indicates that systemic risk relating to the outbreak and development of the COVID-19 pandemic intensifies the risk spillover across cryptocurrency markets (Goodell and Goutte 2020). Other studies identify similar results between cryptocurrencies and stock markets (Conlon and McGee 2020; Corbet, et al. 2020), and cryptocurrency and commodity markets (Dutta et al. 2020).

**Fig. 7:**
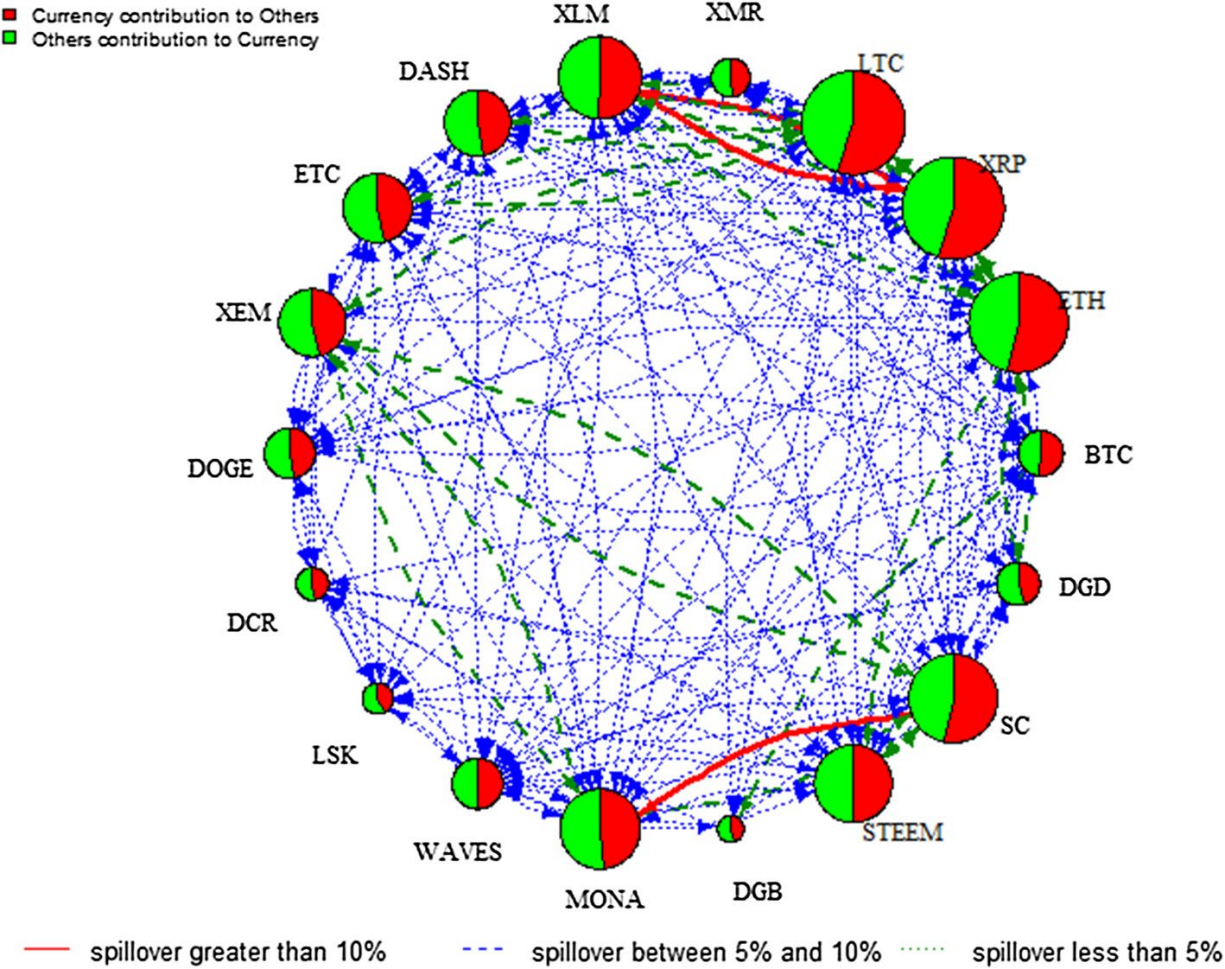
DY Spillover network—low volatility regime of COVID19. *Note*: See notes to Fig. 2

**Fig. 8:**
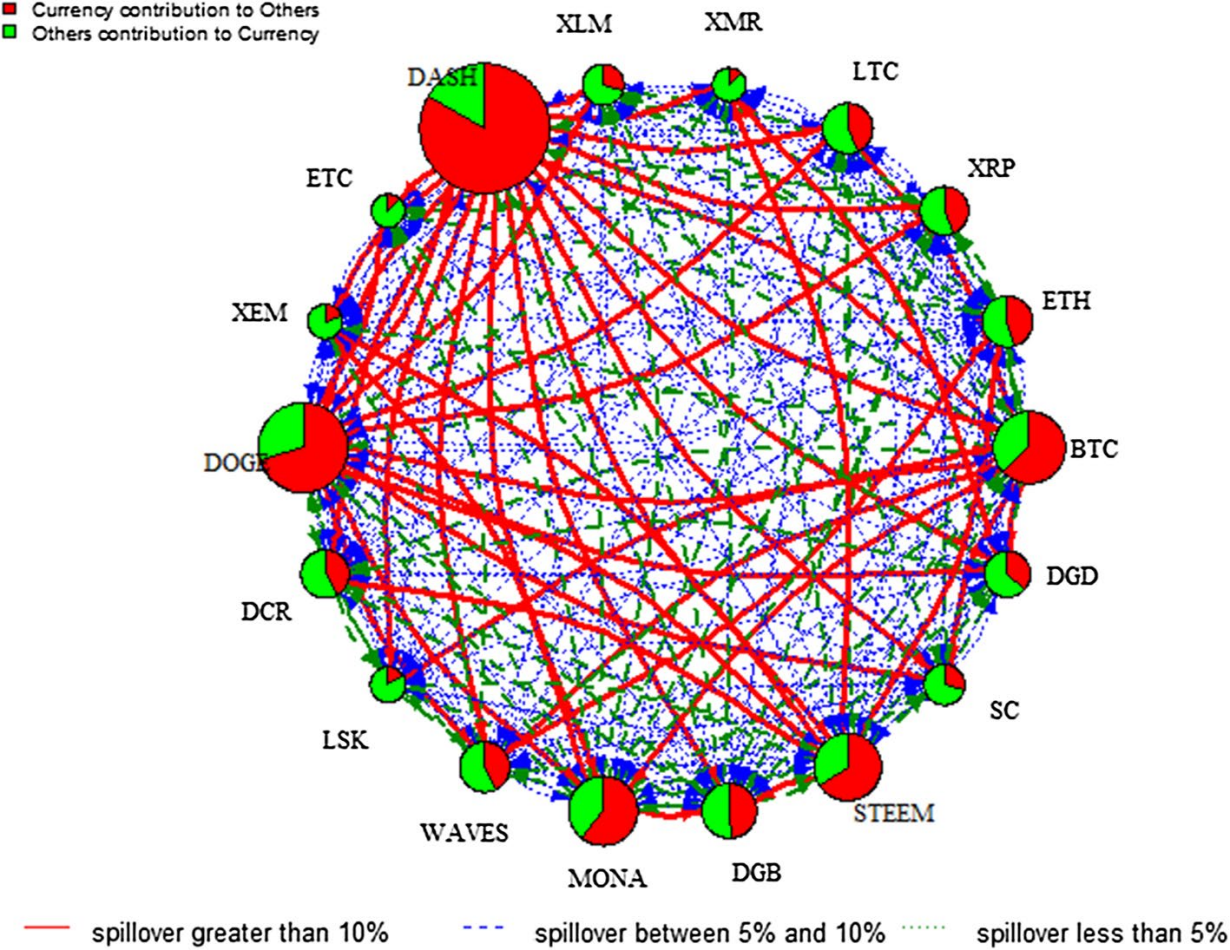
DY Spillover network—high volatility regime of COVID19. *Note*: See notes to Fig. 2

To further analyse the impact of COVID-19, the pairwise directional spillover networks at low and high volatility regimes are given in Figs. 7 and 8, respectively.^8^ The analysis of the COVID-19 outbreak period provides rich information about the intensity and pathway of risk spillover from one cryptocurrency market to another during such an unprecedented catastrophic event. The edge colour denotes the magnitude of the directional spillover and the node diameter denotes the size of the net spillover. Figures 7 and 8 show the complexity of information spillover under various regimes, especially the high volatility regime of COVID-19. Specifically, it is evident that DASH is the largest contributor of spillover in the high volatility regime, whereas strong directional spillover is observed in LTC, XRP, and ETH in the low volatility regime. As indicated in the plots, the directional spillover is much stronger in the high volatility regime than the low volatility regime. This finding confirms that cryptocurrency markets become more dependent in the high-volatility regime. In addition, these networks reflect the way the spillover reacts to the impact of COVID-19 on the regime, to gain a more complex network structure in the high volatility regime. In fact, Table 5 in the “Appendix” shows that in the low volatility regime, the total spillover index is 34.22%, whereas it reaches 96.23% in the high volatility regime. These findings add to previous studies focusing on the effects of specific economic, political, or cybersecurity events on the network of spillovers among leading cryptocurrencies, by showing the significant effect of the COVID-19 outbreak and its subsequent lockdown recession on the network of spillovers, especially in the high volatility regime.

## Conclusion

In this study, we investigate the regime dependent spillovers across 18 cryptocurrency markets in low and high volatility regimes using the MS-VARX model and the spillover measure of Diebold and Yilmaz (2014). In addition, we visualize the dynamics of total spillovers and the complex networks of spillovers in low and high volatility regimes and examine the impact of COVID-19 on the dynamics of spillovers.

The empirical results provide evidence of strong spillovers across the cryptocurrency markets in low and high volatility regimes during the full sample period. However, the spillover effect changes between the regimes with the spillover effect more pronounced in the low volatility regime. The rolling window analysis shows significant structural changes of spillovers in late 2018 and early 2020. Interestingly, the COVID-19 outbreak period in early 2020 amplifies the magnitude of spillovers in the high volatility regime, indicating an increase in the high volatility regimes during the COVID-19 period. Notably, and consistent with the notion of contagion, we find much stronger spillovers across all cryptocurrencies in the high volatility regime during the COVID-19 outbreak period.

Our findings have implications for investors and policymakers. The regime dependent spillover results provide better ways to manage portfolio diversification strategies. In fact, the findings suggest that portfolio diversification opportunities are lower in a high volatility regime. It is necessary for investors to precisely judge which markets they need to be more concerned about, and when, under low and high volatility regimes. Similarly, regime dependent spillovers have implications for policymakers about when to intervene to stabilize markets. Policymakers can use the results to identify the magnitude of spillover in low and high volatility regimes. If necessary, they can intervene and reduce the spike in the spillover effect in a regime by trying to control uncertainties related to stressful periods, announcing definite policy measures capable of elevating the sentiment of the markets and the overall economy. A crucial concern is that, during stress periods such as COVID-19, the return spillovers across leading cryptocurrencies abruptly diverge from their usual paths in an unpredictable way. This matters to policymakers who seek financial stability in financial markets.

Future research could consider portfolio and hedging analysis within the cryptocurrency markets in both regimes and during the COVID-19 outbreak. Another extension could involve the application of a regime-based analysis of spillover periods between the cryptocurrency markets and conventional assets during the COVID-19 period.

## Data Availability

Data will be available from the authors upon request.

## Appendix

**Table 4:** Directional detailed spillovers under regime switching—full sample

	BTC	ETH	XRP	LTC	XMR	XLM	DASH	ETC	XEM	DOGE	DCR	LSK	WAVES	MONA	DGB	STEEM	SC	DGD	From
*Regime 1 (low volatility)*																			
BTC	73.88	1.18	0.89	1.90	0.95	1.62	0.63	0.43	2.03	0.61	0.43	1.15	0.39	6.52	2.61	0.38	3.65	0.76	26.12
ETH	0.46	68.14	0.41	1.64	3.50	1.26	6.25	3.75	2.56	0.60	1.50	1.75	1.82	0.24	0.48	0.14	1.43	4.07	31.87
XRP	0.88	0.67	72.82	1.93	0.57	15.06	1.01	0.17	0.79	3.38	0.01	0.55	0.68	0.11	0.57	0.31	0.38	0.08	27.18
LTC	2.75	1.72	1.05	74.02	2.10	1.35	2.09	2.81	1.60	3.48	0.51	0.67	0.79	0.54	1.21	0.21	1.13	1.98	25.98
XMR	1.54	3.72	0.90	1.72	73.37	3.16	7.04	0.43	1.16	0.26	0.45	0.58	2.19	0.61	0.83	0.41	0.54	1.08	26.63
XLM	1.36	0.43	13.91	0.75	2.04	70.60	0.11	0.15	1.91	4.07	0.15	0.13	0.58	0.08	1.98	0.58	1.12	0.04	29.40
DASH	0.61	5.29	0.61	2.02	6.73	0.06	68.76	3.63	1.33	0.14	3.18	2.73	0.87	0.86	0.50	0.80	1.31	0.57	31.24
ETC	0.66	3.30	0.16	2.88	0.43	0.57	2.68	80.59	0.52	0.90	1.25	1.87	0.67	0.09	0.21	0.11	1.50	1.62	19.41
XEM	2.00	2.54	1.05	1.27	1.38	2.18	1.34	0.86	73.79	0.81	0.99	4.97	1.19	0.95	0.33	2.76	0.75	0.85	26.21
DOGE	0.28	0.57	3.04	3.41	0.30	4.44	0.52	0.53	0.89	72.48	0.21	0.51	0.10	0.17	5.38	1.61	5.50	0.07	27.52
DCR	0.23	0.37	0.68	0.72	0.66	0.82	5.03	0.13	1.27	0.14	80.00	1.38	1.53	0.26	1.80	2.15	2.42	0.40	20.00
LSK	0.82	1.91	1.04	0.42	2.12	0.82	3.84	1.85	3.65	0.30	1.35	71.61	3.58	1.36	0.31	1.94	1.20	1.89	28.39
WAVES	0.62	1.64	0.43	2.33	2.71	0.81	1.45	0.55	0.63	0.21	2.13	3.92	76.02	0.08	1.32	0.86	2.53	1.75	23.98
MONA	0.37	0.28	0.11	0.59	0.63	0.40	1.32	0.32	0.90	0.62	0.27	1.37	0.30	90.87	0.43	0.30	0.12	0.81	9.13
DGB	1.41	0.07	2.17	1.31	0.11	2.71	0.13	0.16	0.14	5.15	2.08	0.25	1.16	0.38	68.65	0.47	13.31	0.35	31.35
STEEM	0.10	0.09	0.29	0.16	0.24	4.09	1.15	0.42	0.86	0.61	2.17	1.95	0.62	0.31	1.08	84.81	0.76	0.28	15.19
SC	1.31	1.26	0.32	0.38	0.47	1.57	1.52	0.81	0.73	5.27	1.34	1.13	0.71	0.59	13.78	1.20	67.11	0.50	32.89
DGD	0.75	5.55	0.11	1.81	1.37	0.06	1.33	1.37	0.72	0.04	1.05	1.73	0.73	0.97	0.09	0.57	0.56	81.18	18.82
To	16.15	30.59	27.19	25.21	26.30	40.99	37.42	18.37	21.70	26.61	19.06	26.66	17.90	14.11	32.91	14.82	38.19	17.12	Total
Net	− 9.97	− 1.27	0.01	− 0.77	− 0.32	11.59	6.18	− 1.04	− 4.51	− 0.91	− 0.94	− 1.73	− 6.08	4.98	1.56	− 0.38	5.31	− 1.71	25.07
*Regime 2 (high volatility)*																			
BTC	82.18	0.98	0.18	5.85	2.43	0.42	2.07	0.53	0.11	0.85	1.32	0.24	0.58	0.33	0.07	0.85	0.67	0.33	17.82
ETH	0.63	55.21	8.62	9.53	3.28	3.89	4.81	3.41	1.14	0.09	0.64	1.31	0.02	0.54	0.95	0.49	1.21	4.24	44.80
XRP	0.32	8.66	51.28	5.70	2.05	10.48	2.72	3.56	2.62	2.18	0.46	1.39	0.18	2.09	1.28	1.11	2.51	1.41	48.72
LTC	3.85	9.41	6.08	54.19	2.36	3.72	3.36	5.02	1.31	1.57	1.51	1.43	0.11	0.55	1.16	0.75	1.64	1.97	45.81
XMR	2.94	3.91	2.74	2.72	66.09	4.19	5.98	1.44	0.79	0.31	0.98	0.96	0.64	0.08	1.02	1.53	2.97	0.71	33.91
XLM	0.03	3.98	10.91	3.63	3.10	52.95	2.48	2.67	2.56	1.44	0.47	2.48	0.32	0.55	3.57	3.24	4.25	1.36	47.05
DASH	1.63	5.65	3.46	3.93	6.03	3.06	62.15	2.84	1.76	0.82	1.28	2.26	0.24	0.90	0.93	0.30	2.12	0.66	37.86
ETC	0.37	4.09	4.64	6.13	1.86	3.39	3.05	66.27	2.15	1.26	0.19	1.57	0.27	1.12	0.19	0.95	1.84	0.67	33.73
XEM	0.08	1.45	3.58	1.69	0.92	3.41	2.01	2.28	70.39	0.59	0.66	1.69	1.17	2.06	1.36	1.06	4.95	0.64	29.61
DOGE	0.83	0.31	3.61	2.37	0.38	2.21	1.17	1.60	0.94	81.59	0.65	0.47	0.24	0.14	1.07	0.05	2.11	0.25	18.41
DCR	0.79	0.75	0.75	1.94	0.84	0.88	1.64	0.57	0.70	0.64	86.19	0.51	0.04	0.99	0.74	1.05	0.67	0.31	13.81
LSK	0.09	1.63	2.00	1.95	1.07	3.27	2.78	1.75	1.74	0.41	0.55	73.87	0.55	0.60	0.84	0.49	4.88	1.52	26.13
WAVES	0.40	0.10	0.19	0.25	0.39	0.55	1.01	0.25	0.64	0.51	0.18	0.57	92.66	0.08	0.39	1.24	0.59	0.01	7.34
MONA	0.28	1.18	3.19	0.89	0.23	1.63	1.17	1.39	2.54	0.31	0.95	0.92	0.43	80.66	0.94	0.46	2.67	0.17	19.34
DGB	0.06	1.29	1.93	1.51	1.09	5.09	1.11	0.23	1.38	0.83	0.71	1.12	0.29	0.91	74.85	1.33	5.48	0.82	25.15
STEEM	0.42	0.77	1.84	1.02	2.59	4.75	0.47	1.16	1.77	0.15	0.98	0.55	1.54	0.37	1.22	77.62	2.74	0.06	22.39
SC	0.18	1.40	3.36	1.72	2.45	5.06	2.09	1.81	4.33	1.69	0.57	4.14	0.36	1.47	4.50	2.05	61.79	1.05	38.21
DGD	0.24	6.02	1.72	1.97	0.93	1.95	0.83	0.79	0.57	0.15	0.41	1.65	0.01	0.16	0.87	0.09	1.27	80.38	19.62
To	13.12	51.57	58.80	52.80	32.00	57.96	38.75	31.31	27.03	13.79	12.52	23.25	7.00	12.94	21.11	17.03	42.55	16.15	Total
Net	− 4.70	6.78	10.08	6.99	− 1.90	10.91	0.89	− 2.42	− 2.58	− 4.62	− 1.30	− 2.88	− 0.34	− 6.40	− 4.04	− 5.35	4.34	− 3.47	29.43

**Table 5:** Directional detailed spillovers under regime switching—COVID-19 period (January 1st, 2020–April 1st, 2020)

	BTC	ETH	XRP	LTC	XMR	XLM	DASH	ETC	XEM	DOGE	DCR	LSK	WAVES	MONA	DGB	STEEM	SC	DGD	From
*Regime 1 (low volatility)*																			
BTC	76.50	0.29	1.33	0.29	3.26	1.33	1.47	0.84	0.17	1.20	0.35	0.06	3.22	1.13	4.09	3.13	1.05	0.30	23.50
ETH	1.40	50.74	9.29	8.20	1.67	5.12	3.95	2.10	0.59	1.52	0.30	0.84	1.38	0.99	0.17	4.65	1.27	5.81	49.26
XRP	0.84	9.16	50.42	6.98	1.42	10.10	2.18	2.72	3.34	1.17	0.53	0.55	0.68	1.79	0.51	2.89	3.41	1.31	49.58
LTC	0.10	8.07	6.59	50.40	1.51	4.41	6.40	6.98	2.97	2.02	0.30	0.27	0.23	1.83	1.28	2.66	3.78	0.22	49.60
XMR	3.42	2.35	2.18	2.47	78.63	1.62	3.79	1.70	0.62	0.54	0.31	0.01	0.37	0.56	0.46	0.18	0.09	0.69	21.38
XLM	1.14	6.16	12.09	5.06	1.42	56.54	1.47	1.21	0.74	0.79	0.80	0.07	3.82	1.16	0.19	2.85	1.84	2.65	43.46
DASH	1.88	4.90	2.82	7.96	2.77	2.75	62.70	4.56	0.05	4.83	0.06	1.30	0.74	1.92	0.34	0.06	0.19	0.17	37.30
ETC	1.30	3.28	4.08	8.52	1.62	1.09	4.35	60.71	1.88	2.11	1.54	0.53	0.68	2.21	0.56	3.10	2.17	0.26	39.29
XEM	0.02	0.33	3.95	5.24	0.59	0.81	0.05	1.51	61.53	0.90	0.73	0.93	2.80	8.19	0.39	2.73	8.75	0.56	38.47
DOGE	2.23	2.69	1.28	2.97	0.99	0.89	4.60	2.47	1.15	71.68	1.83	4.34	0.17	0.64	0.38	0.69	0.69	0.31	28.32
DCR	0.14	0.32	0.83	0.14	0.62	0.61	0.34	2.38	0.13	2.10	81.31	0.71	3.30	3.90	0.19	1.74	0.22	1.01	18.69
LSK	0.04	1.32	0.53	1.03	0.09	0.95	1.70	0.70	1.09	4.92	0.68	80.53	1.27	0.90	0.62	1.09	2.05	0.48	19.47
WAVES	2.25	1.02	1.75	0.91	1.31	4.83	0.55	0.51	0.78	0.28	2.22	0.71	72.99	2.17	1.03	2.83	2.78	1.09	27.01
MONA	1.66	1.08	2.10	2.39	0.70	1.50	1.52	2.32	8.33	0.85	2.54	0.63	2.02	55.97	0.77	3.68	11.04	0.90	44.03
DGB	5.71	0.26	1.05	1.77	0.95	0.28	0.37	0.82	0.27	0.57	0.08	0.40	1.61	0.83	84.14	0.13	0.20	0.56	15.86
STEEM	1.90	5.52	3.46	2.92	0.18	3.01	0.05	2.41	2.82	0.52	2.40	0.45	2.22	3.19	0.44	58.84	7.61	2.07	41.16
SC	0.44	1.59	3.93	3.51	0.38	1.76	0.25	0.86	7.99	0.56	0.29	1.65	2.15	9.22	0.73	6.39	56.17	2.15	43.83
DGD	0.20	8.15	2.31	0.37	0.46	3.63	0.37	0.17	0.55	0.59	1.08	0.23	0.53	0.46	0.37	2.74	3.48	74.31	25.69
To	24.65	56.50	59.54	60.73	19.94	44.69	33.39	34.28	33.45	25.50	16.05	13.68	27.18	41.09	12.54	41.52	50.64	20.54	Total
Net	1.15	7.24	9.97	11.13	− 1.44	1.22	− 3.91	− 5.01	− 5.02	− 2.82	− 2.64	− 5.79	0.17	− 2.94	− 3.33	0.36	6.81	− 5.15	34.22
*Regime 2 (high volatility)*																			
BTC	12.10	5.84	4.38	5.13	0.61	1.80	26.74	0.47	0.49	17.61	3.17	0.87	1.04	9.36	6.29	1.41	2.31	0.40	87.90
ETH	2.88	8.62	7.89	2.29	1.74	3.60	7.49	1.03	1.86	3.93	3.84	0.79	16.44	2.95	2.16	22.35	1.08	9.08	91.38
XRP	6.07	9.32	10.68	3.89	0.93	5.16	14.60	0.80	0.96	18.57	1.31	1.44	8.15	8.91	4.88	2.52	1.40	0.41	89.33
LTC	11.76	4.79	3.47	5.72	0.57	1.51	27.43	0.48	0.79	15.69	3.76	0.69	1.05	8.84	6.09	3.44	2.68	1.23	94.28
XMR	4.80	4.21	3.74	3.26	1.82	1.99	12.54	1.19	1.30	6.43	4.96	0.74	3.96	5.40	4.39	26.82	0.85	11.58	98.18
XLM	9.21	6.98	6.20	4.24	0.96	3.29	19.89	0.67	0.64	16.80	2.67	1.00	3.04	8.96	5.36	6.01	1.67	2.43	96.71
DASH	12.19	5.57	3.83	5.13	0.57	1.55	28.63	0.38	0.49	17.61	3.33	0.76	0.74	9.50	6.51	0.46	2.44	0.31	71.37
ETC	7.32	8.05	7.57	3.85	0.92	3.15	17.26	3.91	0.28	18.30	1.88	1.24	5.47	7.19	5.75	4.14	1.05	2.69	96.09
XEM	4.57	2.45	2.99	3.44	1.06	3.73	15.97	1.87	7.01	3.20	4.11	3.85	6.42	10.71	3.36	14.45	3.51	7.30	92.99
DOGE	9.03	1.81	4.28	4.50	0.67	2.13	18.28	1.69	2.34	11.06	5.53	1.20	3.22	7.54	3.98	16.44	2.65	3.65	88.94
DCR	8.59	3.15	3.15	4.32	0.67	1.64	18.97	1.09	1.47	7.81	6.27	0.73	3.01	5.22	5.02	19.44	2.00	7.44	93.73
LSK	11.39	1.14	1.59	4.10	0.43	1.26	30.80	0.65	2.96	6.36	5.31	2.06	6.44	9.94	4.53	6.15	3.68	1.21	97.94
WAVES	13.57	2.35	1.42	4.96	0.32	0.81	32.60	0.29	1.22	10.98	5.18	0.48	3.75	8.47	5.68	4.12	3.33	0.48	96.25
MONA	12.76	4.23	2.64	4.90	0.60	1.07	29.84	0.40	1.09	15.47	3.77	0.71	1.01	10.17	6.01	1.64	2.97	0.73	89.83
DGB	7.71	3.83	3.66	4.45	0.65	3.60	18.74	1.08	3.09	9.65	4.68	2.12	3.11	11.75	6.19	10.28	2.91	2.50	93.81
STEEM	6.70	3.86	6.82	3.94	1.49	4.04	12.43	0.72	1.34	9.00	4.81	1.52	4.04	6.34	5.47	23.64	1.15	2.70	76.37
SC	10.47	6.17	4.88	5.20	0.72	2.35	24.65	0.64	0.72	17.53	3.08	1.18	1.69	9.19	6.12	0.92	3.77	0.70	96.23
DGD	12.12	3.32	2.27	4.96	0.62	1.22	29.40	0.39	1.08	12.92	4.51	0.54	1.17	9.05	5.95	5.63	2.81	2.04	97.96
To	151.12	77.07	70.78	72.56	13.55	40.61	357.63	13.84	22.11	207.85	65.90	19.87	70.00	139.30	87.55	146.21	38.47	54.85	Total
Net	63.22	− 14.31	− 18.54	− 21.72	− 84.63	− 56.10	286.26	− 82.25	− 70.87	118.91	− 27.83	− 78.07	− 26.24	49.47	− 6.26	69.84	− 57.75	− 43.11	91.63

## Footnotes

1 These include economic policy uncertainty, geopolitical risk, stock market volatility, and macroeconomic news, among other variables (Al-Khazali et al. 2018; Demir et al. 2018; Aysan et al. 2019; Bouri et al. 2020a).

2 Chaim and Laurini (2019) examine the joint dynamics of cryptocurrencies and point to shifts in cryptocurrencies return dynamics.

3 Some recent studies point to the role US Treasury Securities as a safe haven asset during the COVID-19 outbreak.

4 A related approach is found in Malugin and Novopoltsev (2017). Other studies (Kou et al. 2014; Wen et al. 2019; Wang et al. 2020; Chao et al. 2020; Shen et al. 2020b) apply interesting methods that can be the subject of future research.

5 The R package “crypto” is used to access the data. See https://www.rdocumentation.org/packages/crypto/versions/1.1.3 for details.

6 These figures are based on Table 4.

7 The regime shifted from low to high on January 14th, 2020.

8 These networks are based on Table 5.
